# Methadone vs. morphine SR for treatment of neuropathic pain: A randomized controlled trial and the challenges in recruitment

**DOI:** 10.1080/24740527.2019.1660575

**Published:** 2019-10-22

**Authors:** Mary Lynch, Dwight Moulin, Jordy Perez

**Affiliations:** a Anesthesia, Pain Management and Perioperative Medicine, Psychiatry and Pharmacology, Dalhousie University, Halifax, Nova Scotia, Canada; b Department of Clinical Neurological Sciences, Western University, London, Ontario, Canada; c Department of Anesthesia and Perioperative Medicine, Western University, London, Ontario, Canada; d Department of Anesthesia, Alan Edwards Pain Management Unit, McGill University Health Centre (MUHC), Montreal, Quebec, Canada

**Keywords:** neuropathic pain, opioids, methadone, study recruitment

## Abstract

**Introduction**: Accumulating evidence has identified a number of advantages for methadone over other opioids for the treatment of chronic pain including: agonist action at both μ and δ opioid receptors, *N*-methyl-d-aspartate (NMDA) antagonist activity and the ability to inhibit the reuptake of monoamines. It was hypothesized that with these three mechanisms of action methadone might be a good option for the treatment of neuropathic pain.

**Methods**: This was a double-blind randomized controlled trial comparing methadone to controlled-release morphine. The primary objective was to determine whether methadone is clinically inferior versus noninferior to morphine as an analgesic.

**Results**: We attempted recruitment at three academic pain centers over a 3-year period. In the end only 14 participants were able to be recruited; 5 withdrew and only 9 completed the trial. This study was underpowered. All participants showed a mean reduction in pain intensity according to the Numeric Rating Scale for Pain Intensity (morphine 5.86 to 4.38, methadone 6.11 to 4.5) and reported pain relief compared to pretreatment, but the sample size was too small for statistical analysis.

**Discussion**: Reasons for challenges in recruitment included tight inclusion and exclusion criteria and high participant burden. In addition, there was significant heterogeneity of patients between the three sites, leading to differences in reasons for exclusion. This included seemingly disparate reasons at the different sites, including few participants who were methadone naïve vs. avoidance or fear of opioids. In the end, we were unable to answer the question of the study.

## RÉSUMÉ

**Introduction**: L'accumulation de données probantes a permis de répertorier un certain nombre d'avantages pour la méthadone comparativement à d'autres opioïdes pour le traitement de la douleur chronique, dont l'action agoniste sur les récepteurs opioïdes μ et δ, l'action antagoniste du N-méthyl-D-aspartate (NMDA) et la capacité d'inhiber la recapture des monoamines. L'hypothèse émise était qu'avec ces trois mécanismes d’action, la méthadone pourrait être une bonne option pour le traitement de la douleur neuropathique.

**Méthodes**: Il s'agissait d'un essai à double insu, randomisé et contrôlé comparant la méthadone à la morphine à libération contrôlée. L'objectif principal était de déterminer si la méthadone est cliniquement inférieure ou non inférieure à la morphine comme analgésique.

**Résultats**: Nous avons tenté de recruter dans trois centres universitaires de la douleur sur une période de trois ans. Au final, seulement 14 participants ont pu être recrutés, parmi lesquels cinq se sont retirés, de sorte que seulement neuf ont terminé l'essai. La puissance de l’étude était donc insuffisante. Tous les participants ont montré une réduction moyenne de l'intensité de la douleur selon l'échelle numérique de notation de l'intensité de la douleur (morphine 5,86 à 4,38, méthadone 6,11 à 4,5) et ont signalé un soulagement de la douleur par rapport au prétraitement. Cependant, la taille de l'échantillon était trop petite pour une analyse statistique.

**Discussion**: Les difficultés de recrutement ont été attribuées à des critères d'inclusion et d'exclusion étroits et à un fardeau élevé pour les participants. En outre, il y avait une hétérogénéité importante parmi les patients d’un emplacement à l’autre, de sorte que les raisons pour l’exclusion étaient différentes. En effet, des raisons apparemment disparates ont été répertoriées aux différents sites, dont le faible nombre de participants n’ayant pas de tolérance acquise à la méthadone, l'évitement ou la peur des opioïdes. En fin de compte, nous n'avons pas été en mesure de répondre à la question de l'étude.

## Introduction

This study was prompted by accumulating evidence that methadone has a number of advantages over other opioids for the treatment of chronic pain, including agonist action at both μ and δ opioid receptors,^1^ *N*-methyl-d-aspartate (NMDA) antagonist activity,^2,3^ and the ability to inhibit the reuptake of monoamines.^4^ Pharmaco-economic issues related to the very low cost of generic hydrochloride methadone powder also led to increased use of methadone for the treatment of cancer pain,^5^ neuropathic pain,^6,7^ and non-cancer pain.^8,9^

The NMDA antagonist effect led to speculation that methadone may be a better opioid for the treatment of neuropathic pain.^6^ NMDA receptors, members of the ligand-gated ion channel superfamily of glutamate receptors, are known to exhibit minimal activity within pain systems under normal physiological conditions but subsequent to injury have been implicated in pain processing, with generation and maintenance of central hypersensitivity contributing to chronic pain.^10–12^ NMDA antagonists have also been demonstrated to prevent the development of opioid tolerance.^13,14^ One study confirmed that d-methadone blocks morphine tolerance and NMDA-induced hyperalgesia in animal models.^3^

Methadone has also been demonstrated to inhibit 5-hydroxytryptamine (serotonin) and noradrenaline uptake and the antinociceptive activity of methadone had been found to be related to both opioid and monoamine uptake activity,^4^ whereas phenanthrine opioids such as codeine and morphine do not block 5-hydroxytryptamine and noradrenaline uptake.

The advantage of additional mechanisms of action for neuropathic pain raises the question of whether methadone might be a better option than conventional opioids when clinicians think that a trial of an opioid is appropriate. In the field of cancer pain management, this option has been suggested, although the literature is inconclusive.^15^ At the time we planned this study, there were no published controlled trials examining methadone for the treatment of neuropathic pain, and we thought it was important to subject this to further study. Since then, there have been two Cochrane reviews,^16,17^ each involving three studies. One found limited evidence of the effectiveness of methadone for chronic non-cancer pain. The results could not be combined statistically and there were too few participants in each study to be confident in the results.^16^ The second found very limited, low-quality evidence of the efficacy and safety of methadone in the treatment of chronic neuropathic pain.^17^ The current clinical trial was originally designed to examine efficacy and safety of methadone compared to a conventional opioid morphine slow-release in the treatment of neuropathic pain.

## The original plan and challenges

The design involved examining methadone against an established “gold standard” treatment, in this case slow-release morphine, in the management of neuropathic pain. We wanted to know whether methadone was at least as good as morphine and designed the trial to test whether it was clinically inferior versus noninferior to morphine.

## Power, sample size, and statistical plan

In a previous opioid randomized controlled trial of similar design, with similar subjects, using a pain scale that also ranged from 0 to 10, the standard deviations of the pain scores (between subjects) in the two treatment arms were 1.82 and 1.70, respectively, at baseline, and 2.65 and 2.47, respectively, at the final dose.^18^ We therefore estimated that the standard deviation of the pain reduction from baseline to stabilization (within subjects) is no more than 2.5. A one-sided two-sample *t* test (comparing mean reductions in the two treatment arms) at the alpha = 0.025 level of significance based on 45 subjects per treatment arm has 96% power to reject the inferiority of methadone, where inferiority is defined as a difference of 2 points on the pain scale. We planned 67 subjects per treatment arm to provide increased confidence and enable us to adjust for site differences and other potential confounders. The primary analysis was to be a head-to-head comparison of methadone versus morphine using a one-sided 95% confidence interval for the difference in pain score reduction adjusting for baseline characteristics. Methadone was to be deemed noninferior if the confidence interval lies entirely to the right of −2, consistent with the decision that a 2-point difference in pain score represents a minimal clinically significant difference.

## Methods

This study was designed and is being reported according to the CONSORT guidelines for randomized clinical trials (http://www.consort-statement.org). The study consisted of a double-blind randomized controlled trial comparing methadone with controlled release morphine.

The specific primary objective was to determine whether methadone is clinically inferior versus noninferior to morphine in terms of the pain relief that it provides when administered under tightly controlled self-titrating conditions using an 11-point numerical rating scale for pain intensity. The secondary objectives were to assess safety and side effect profiles and to further investigate the equi-analgesic dose of methadone relative to morphine in treatment of neuropathic pain. This study was approved by the ethics review committees at all study sites (Halifax ROMEO File No. 1,002,228, Montreal, 14-179-BMD, London Western REB 17,478), and all participants gave full informed consent to take part in the study. The study was registered at clinical trials.gov (NCT01205516).

### Participating sites

The study was conducted at three academic Canadian sites. Before recruitment started, one of our sites dropped out. We enlisted another interested site and the study was ultimately conducted in academic pain clinic sites in Halifax, London, and Montreal. Recruitment was a challenge related to different issues at each of the sites. One site had a dedicated clinic for neuropathic pain and at that site many patients did not meet the inclusion criteria due to the fact they were already on too high a dose of opioid to qualify for the trial (maximal dose for inclusion was 90 mg/day morphine mg equivalents at study start). Midway through the study, due to challenges in recruitment, an amendment was submitted to expand the inclusion criteria to allow patients on doses up to 160 mg/day oral morphine equivalents to take part in the trial. This was approved, but in the meantime there was a significant change in the sociopolitical climate regarding the use of opioids, which will be presented in the Discussion.

## Randomization and blinding

A randomization schedule was prepared by the study biostatistician and was provided to the study pharmacy where the study medication was prepared. This schedule was computer generated and was done in three blocks such that each study site would have an equal number of participants assigned to each treatment. The off-site study pharmacy packaged the medications, assigned participant numbers according to this randomization list, and shipped the study medication to the appropriate pharmacy at each of the study sites. In this way, study personnel and patients were completely blinded regarding the study medication. They only knew the participant study number.

## Patient population

The study involved participants with moderate to severe chronic neuropathic pain of central or peripheral origin present for 3 months or longer. Inclusion and exclusion criteria are listed in Table 1.

## Drug formulation

### The medications

Patients in the methadone arm were supplied with blinded capsules containing 2.5 mg of methadone. The dose consisted of 1 to 12 capsules taken twice daily, every 12 hours (range 5–60 mg per 24 hours). The comparator consisted of controlled-release morphine supplied with blinded 10 mg capsules, 1 to 12 capsules taken twice daily, every 12 hours (range 20–240 mg per 24 hours). The methadone and morphine capsules were indistinguishable and were prepared off-site at the study pharmacy located in Moncton, New Brunswick. The prepackaged capsules were shipped to each of the study locations in containers with labels that included the participant number and no medication name. Once randomized, participants were dispensed the study medication by the study pharmacist according to their participant number.

## Dosage regimen

In view of the uncertain potency ratio between methadone and conventional opioids, the dosing protocol allowed titration to a point where the pain reduction was maximized without side effects becoming troublesome to the patient. Participants were instructed to start with a dose of one capsule every 12 hours. Within limits of safety and tolerability, participants gradually increased the 12-hourly dose by one to two capsules every second day such that by the end of 35 days they were allowed a maximum of 24 capsules per 24 hours (12 capsules every 12 hours). The goal of the titration phase was to reach a target dose that maximized pain reduction without causing troublesome side effects. This process was similar to that used when titrating opioid doses in the pain clinic.

### Duration of treatment

The dose titration phase took place over a 5-week (35 day) period and treatment continued for six more weeks, allowing 2 weeks for the pain to stabilize and 4 weeks to maintain a steady state, totaling 77 days (11 weeks) of treatment.

## Study activities

Participants attended the clinic on seven occasions over the course of the trial with phone contact in between to address any questions and inquire about side effects.

### Primary outcome measure

In accordance with the Initiative on Methods, Measurement, and Pain Assessment in Clinical Trials (IMMPACT), outcome measures included assessment in several core domains, the first of which was pain.^19,20^ Pain was measured using an 11-point Numerical Rating Scale for Pain Intensity (NRS-PI) with anchors at 0 (*no pain*) and 10 (*pain as bad as you can imagine*). Numerical rating scales have been widely used in pain research and have been demonstrated to be capable of identifying clinically meaningful changes.^21^

### Secondary outcomes

Secondary outcome measures included measures in the remaining domains suggested by IMMPACT.^20^ These included the following:

- Physical functioning: Brief Pain Inventory (BPI) Pain Interference Scale
- Quality of Life (SF12)
- Emotional functioning: Profile of Mood States (POMS)
- Patient Global Impression of Change (PGIC) and Patient Satisfaction Scale

***Physical functioning:*** The BPI Pain Interference Scale has been widely used and found to provide a reliable and valid measure of pain’s interference with physical functioning in seven areas, including general activity, mood, walking ability, work, relations with other people, sleep, and enjoyment of life.^22^ The instrument consists of a series of 11-point numeric rating scales asking the participant to indicate how much the pain has interfered with these seven areas (0 = *does not interfere*, 10 = *completely interferes*). The instrument has been translated into many languages and studied in diverse chronic pain conditions in many countries.^22^

***Quality of life:*** The SF12 is a reliable and valid shortened version^23^ of the SF-36 Health Survey,^24^ which is the most commonly used generic measure of health-related quality of life.

***Emotional functioning:*** Chronic pain is often accompanied by symptoms of psychological distress. IMMPACT has recommended the POMS as a good core measure to assess the major aspects of emotional functioning in chronic pain clinical trials.^20^ The POMS has well-established reliability and validity in the assessment of symptoms of emotional distress and has been used in numerous clinical trials in psychiatry and chronic pain. The POMS assesses six mood states (tension-anxiety, depression-dejection, anger-hostility, vigor-activity, fatigue-inertia, and confusion-bewilderment) and also provides a summary measure of total mood disturbance.

***Global impression of change and satisfaction:*** Global ratings or improvement and satisfaction provide an opportunity for participants to rate the agent in one overall measure that conveys his or her perception of the advantages or disadvantages of the treatment received.^20^ IMMPACT has recommended the PGIC. This measure is a single-item rating by participants of their improvement with treatment on a 7-point scale that ranges from *very much improved* to *very much worse*, with *no change* as the midpoint.^25^ The Patient Global Satisfaction Scale is a 10-point scale with verbal descriptors ranging from *very satisfied* to *not at all satisfied*.

## Concomitant medication

Participants using concomitant nonopioid analgesics (e.g., nonsteroidal anti-inflammatory drugs, anticonvulsants, antidepressants, and acetaminophen) were permitted to continue use of these medications unchanged during the course of the study as long as doses were stable for 14 days prior to entering the trial. Potential additive side effects such as sedation, fatigue, dizziness, or light-headedness were monitored. Medication types and dosages were recorded and changes in concomitant medications were monitored during the trial. The use of another opioid was not permitted during the trial.

## Results

**Figure 1.**
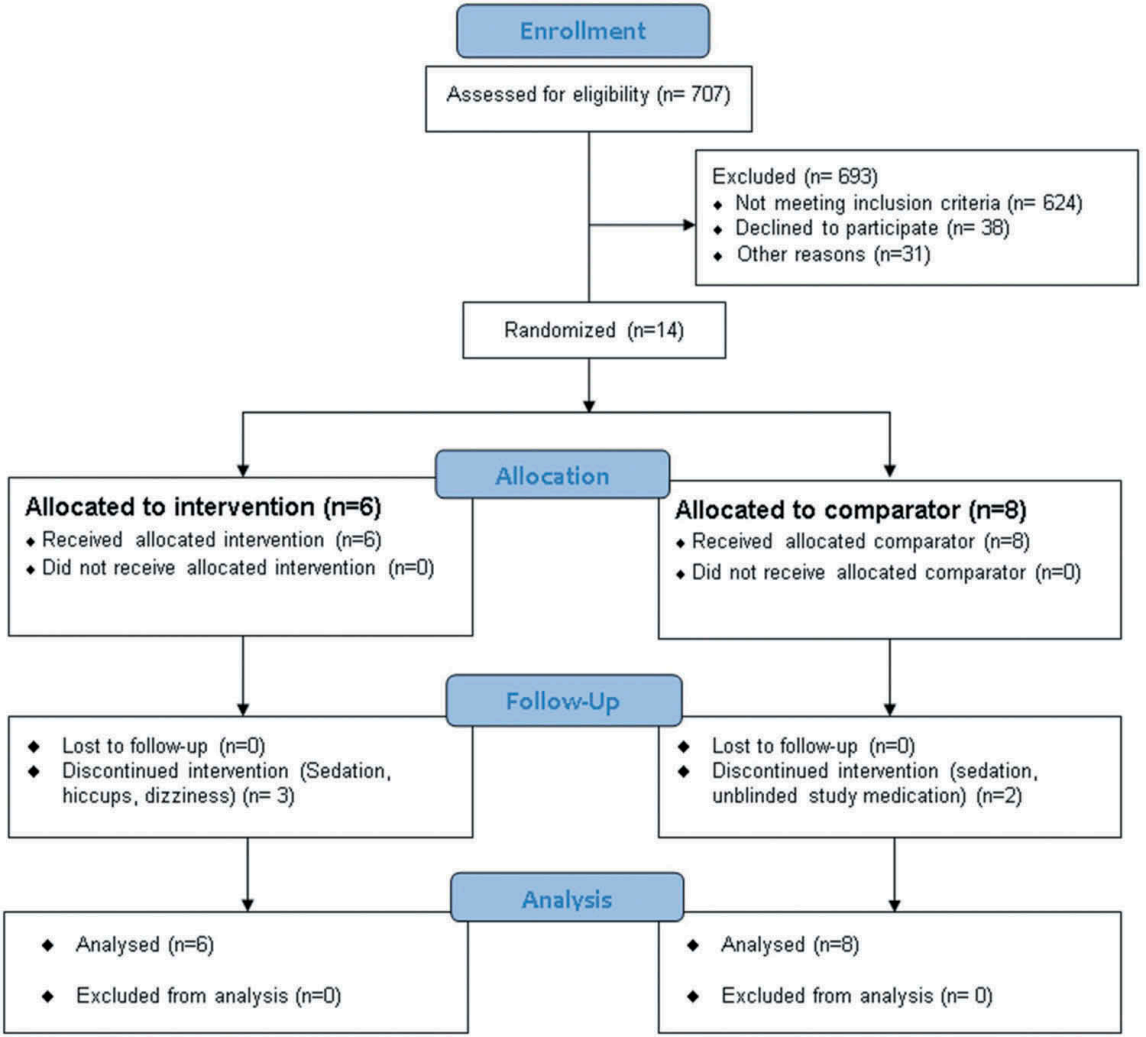
Flow diagram of the study selection process.

Participation in the study across three centers took place over 36 months (March 2012 to March 2015). The London, Ontario, site had a dedicated neuropathic pain clinic, and the other two sites received referrals for any type of chronic pain. Detailed information about participant flow and study exclusions was recorded at all sites and is presented in the CONSORT flow diagram (Figure 1) and Table 2. Table 3 presents information regarding reasons for nonrecruitment according to study site, which demonstrates a major heterogeneity of patients between the three sites. For example, at site 3 methadone was routinely used in neuropathic pain, so there were very few who were methadone naïve; at site 2 many patients had already had a trial of an opioid in doses above 90 mg/day morphine mg equivalents; and at site 1 there was more of an avoidance or fear of opioids.

**Table 1.** Inclusion and exclusion criteria.

Inclusion criteria	Exclusion criteria
Age ≥ 18 years Chronic neuropathic pain of central or peripheral origin for 3 months or longer as determined by the study physician and a score of 4/10 or greater on the DN4^a^	Patients who have never been on opioid therapy Patients on a dose of opioid that exceeds 90 mg/24 hours in OME (a protocol amendment increased the threshold to to 160 mg OME due to challenges in recruitment)
Moderate to severe pain as defined by average 7-day pain score of greater than 4 on an 11-point Numerical Rating Scale for Pain Intensity Physician has identified that an opioid is a valid adjunctive treatment for the chronic neuropathic pain	Patients who have cancer or cancer currently in remission Presence of severe pain disorder other than the chronic neuropathic pain under study that would interfere with patient’s ability to determine effect of study treatment on the chronic neuropathic pain
Concomitant nonopioid analgesic medications must have been stable for 14 days	Pregnant or lactating women (women of childbearing potential must have negative pregnancy test)
Co-interventions such as TENS, acupuncture, and massage must have been stable for 14 days prior to the trial	History of psychosis History of (within the past 2 years) or current substance dependency disorder
If taking an opioid, maximum dose of opioid in OME is 90 mg/24 hours	Patient taking an excluded medication or with a history of opioid allergy
Ability to follow the protocol with reference to cognitive and situational conditions; for example, stable housing, able to attend follow-up visits	Presence of clinically significant cardiac or pulmonary disorder on physical exam that would compromise participants’ safety in the trial as judged by the study physician
	Participation in another clinical trial in the 30 days prior to enrollment
	Abnormalities above 1.5 times upper range of normal on screening CBC, blood chemistry including BUN, Cr, LDH, AST, ALT
Willing and able to give written informed consent	Presence of significant conduction delay, ischemia, or arrhythmia on screening ECG
	Participation in another clinical trial in the 30 days prior to enrollment

^a^The DN4 consists of a 10-item scale that includes a series of qualitative descriptors of pain as well as sensory examination findings known to be associated with neuropathic pain. The DN4 has been validated in patients with neuropathic pain and has been found to exhibit a sensitivity of 78.0% and a specificity of 81.2%. A score of 4/10 or greater is associated with a diagnosis of neuropathic pain.^26^

DN4 = neuropathic pain diagnostic questionnaire; OME = oral morphine equivalents; TENS = transcutaneous electrical nerve stimulation; CBC = complete blood count; BUN = blood urea nitrogen; Cr = creatinin; LDH = lactate dehydrogenase; AST = aspartate transaminase; ALT = alanine aminotransferase; ECG = electrocardiogram.

**Table 2.** Number of patients screened and reasons for exclusion.

	Site 1	Site 2	Site 3
Age < 18 years	0	0	0
Not primarily neuropathic pain	10	73	198
Pain less than 4/10	8	32	33
Cognitive/behavioral issues (including psychosis)	0	25	10
Situational (unable to travel, moving away)	0	61	15
Opioid dose > 90 mg OME/day	3	59	7
Substance abuse last 2 years	0	9	0
Excluded co-medication	0	1	1
Allergy or significant adverse effect to opioid previously	5	12	4
Declined consent, no specific reason	5	4	5
Already on methadone or failed trial morphine	8	33	15
Other	60^a^	0	11
Total	99	309	299

^a^Did not think the drug was right for them or tried in past with no results, did not want to start an opioid or fear of side effect of drowsiness (14); family doctor influence (6); not interested or did not return calls (23); did not meet one of exclusion criteria other than those listed (17).

OME = oral morphine equivalents.

**Table 3.** Differences between sites in reasons for nonrecruitment.

	Site 1	Site 2	Site 3
Cause 1	Not interested/not returned calls 23 patients (23.2%)	Not primarily neuropathic pain 73 patients (23.6%)	Not primarily neuropathic pain 198 patients (66.2%)
Cause 2	Meeting other exclusion criteria 17 patients (17.2%)	Situational (unable to travel, moving away) 61 patients (19.7%)	Pain less than 4/10 33 patients (11%)
Cause 3	Did not think the drug was right for them or tried in past with no results, did not want to start an opioid or fear of side effect of drowsiness 14 patients (14.1%)	Opioid dose > 90 mg OME/day 59 patients (19.1%)	Already on methadone or failed trial morphine 15 patients (5.0%)

OME = oral morphine equivalents.

Difficulty with patient recruitment led to a lack of statistical power and failure to reach a conclusion in terms of methadone’s inferiority/noninferiority. A purely descriptive analysis is presented in this article. Mean pain scores at each clinic visit from baseline to stable dose are plotted in each of the two analgesic groups. Similar plots have been constructed showing the trend in the mean values of other key outcome quantitative variables.

After 3 years, only 14 participants were randomized into the trial and the study was stopped due to lack of recruitment within the funding time allowed. Five participants withdrew at some point during the study due to adverse events. The study was therefore underpowered and we could not demonstrate noninferiority. Reasons for withdrawal, demographic information, pain diagnosis, concurrent medications, and study status are shown in Table 4. The majority of participants reported at least one adverse effect, the most common of which were fatigue, low energy, constipation, and decreased libido. The final stable dose of opioid in those who completed the trial was a mean of 19.5 mg/day (12.5–37.5 mg) for methadone and 127 mg/day (80–200 mg) for sustained-release morphine.

**Table 4.** Information regarding participants in the study.

Patient number	Age	Sex	Diagnosis	Duration pain (months)	Co-analgesic drugs during study	Prestudy opioid dose/day	Study status
10-001	75	F	Postherpetic neuralgia	16	Gabapentin	0	Withdrew at visit 7 (sedation)
10-002	71	M	Lumbar radiculopathy	4	0	Tramadol 975 mg	Completed
10-003	54	F	Cervical/thoracic radiculopathy	96	0	Fentanyl 12 + HM 6 mg	Completed
10-006	51	F	Cervical radiculopathy	60	Celebrex	HM 12 mg	Completed
10-009	79	M	Neuropathic foot pain post-chemotherapy	48	0	Tramadol 37.5 mg	Withdrew at visit 3 (hiccups)
10-011	60	F	Intercostal neuralgia	30	0	Tramadol 150 mg	Completed
10-013	53	M	Cervical thoracic radiculopathy	84	Amitriptyline	0	Completed
10-14	45	M	Lumbar radiculopathy	132	Acetaminophen Ibuprofen	0	Withdrew at visit 4 (sedation)
10-15	70	M	Posttraumatic neuropathic foot pain	150	Pregabalin	Morphine 120 mg	Completed
10-17	42	F	Diabetic neuropathy	24	Gabapentin	Tylenol 1	Completed
10-18	49	M	Lumbar radiculopathy	24	Pregabalin Cannabis	0	Withdrew at visit 5 (looked inside capsule)
20-001	73	F	Diabetic neuropathy	108	Nabilone	0	Withdrawn at visit 3 (confusion, admitted to hospital)
20-002	43	F	Back and arm pain due to syrinx	52	Nabilone Duloxetine Gabapentin	Oxycocet 3/day	Completed
30-002	60	M	Postsurgical neuropathic	89	Venlafaxine	Butrans 5 μg/h	Completed

HM = hydromorphone.

**Figure 2.**
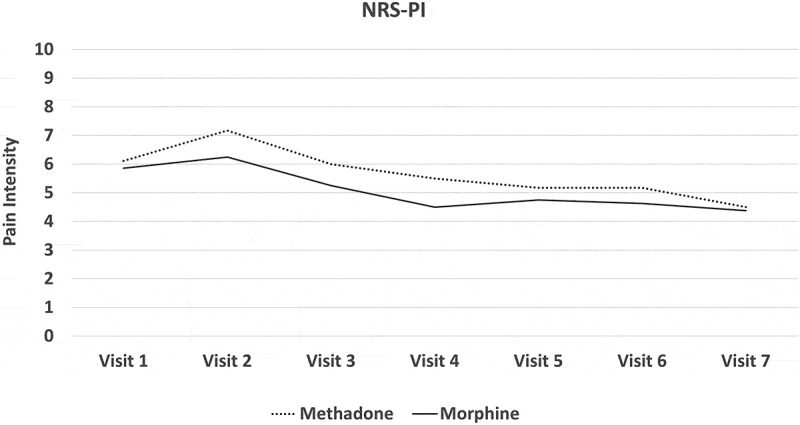
Numeric Rating Scale—pain intensity.

**Figure 3.**
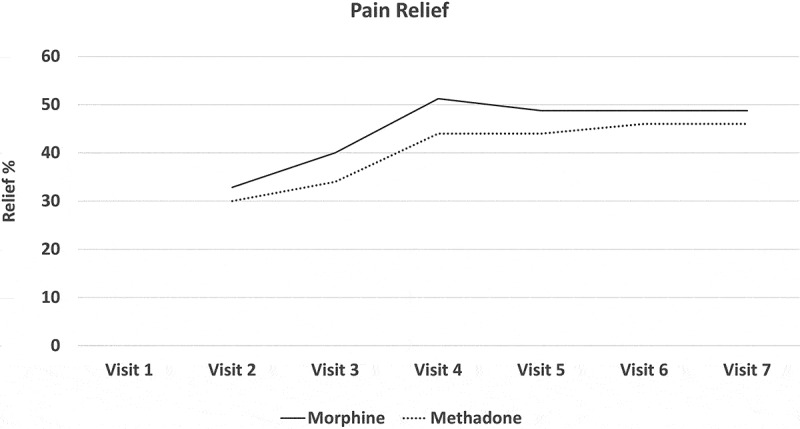
Pain relief score.

**Figure 4.**
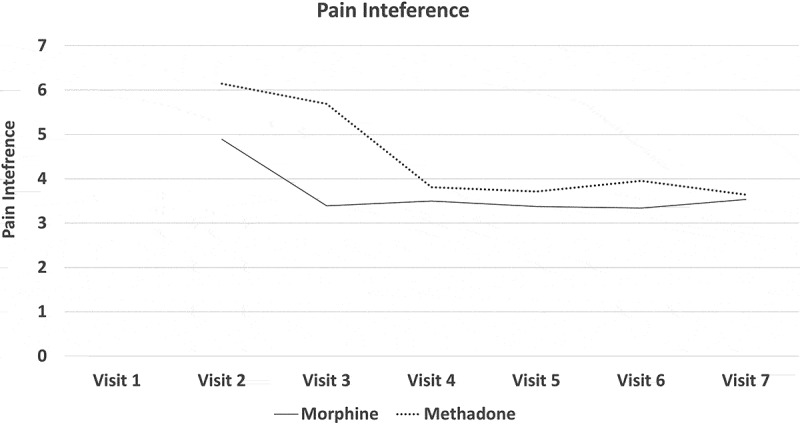
Pain interference score on the brief pain inventrory.

**Figure 5.**
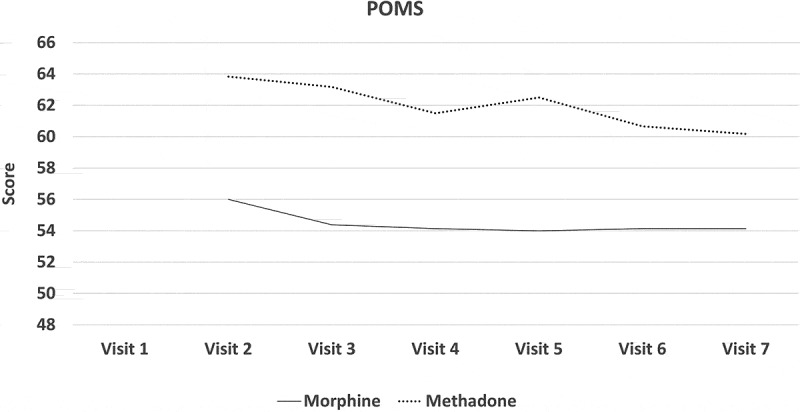
Profile of mood states scores.

**Figure 6.**
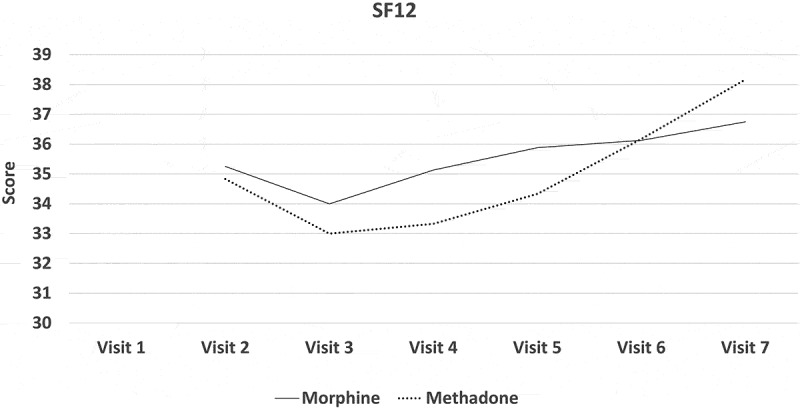
Score on the SF-12 Health Survey.

**Figure 7.**
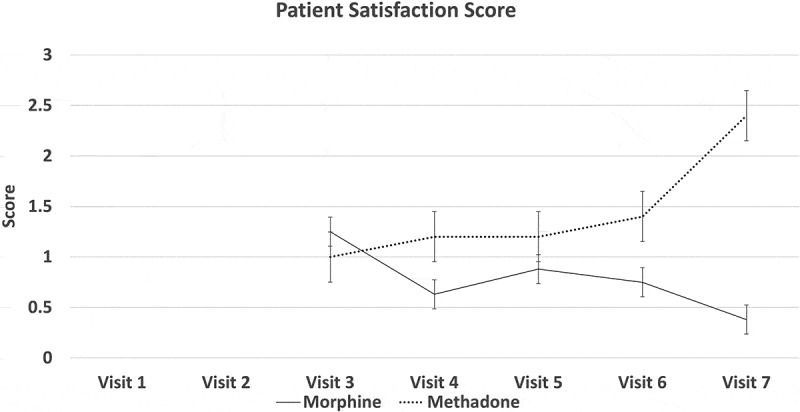
Patient satisfaction score.

**Figure 8.**
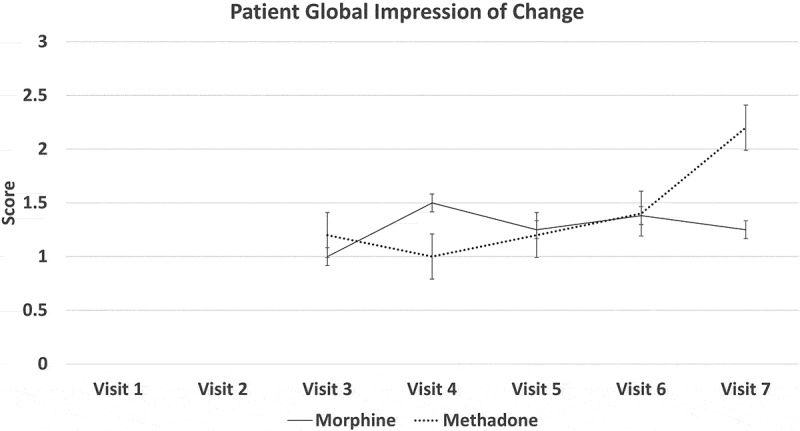
Score on patient global impression of change.

All participants experienced a mean reduction in pain intensity relative to pretreatment. In the morphine arm, the mean NRS-PI decreased from 5.86 to 4.38. In the methadone arm, it decreased from 6.11 to 4.5. Numbers were too small to assess the noninferiority of methadone or even to determine superiority of either drug by means of conventional statistical tests of significance. Figures 2–8 Figure 2 through Figure 8 present the NRS-PI, pain relief, BPI interference, POMS, SF12, Patient Satisfaction Scale, and PGIC data for both groups. Patient satisfaction and global impression of change (visit 7) were better with methadone than with morphine by the end of the study.

## Discussion

Our difficulties in recruitment for this clinical trial reflect the demands of this study. This included relatively tight inclusion and exclusion criteria and demands on the participants as well as potential concerns around opioids. As presented in Table 2, for example, patients who declared that chronic neck or back pain was greater than radicular extremity pain had to be excluded because they did not have primarily neuropathic pain. In addition, the necessity of making seven study visits over 16 weeks to a tertiary center was also challenging for patients who often report increased discomfort with traveling.

Although this study was underpowered to determine whether methadone was noninferior to morphine in treatment of neuropathic pain, interesting data did emerge.

Both morphine and methadone were associated with a reduction in pain and reports of relief. Final mean stable doses of medication were 19.5 mg/day of methadone (range 12.5–37.5 mg) and 127 mg/day of morphine (range 80–200 mg/day). Both opioids were associated with similar rates and types of side effects, with tiredness and low energy being the most frequent. Close to 30% withdrew before completing the study due to adverse effects, which is consistent with other opioid studies. There was a trend for patient global impression of change in pain and satisfaction levels to be higher with methadone than with morphine.

## The opioid pendulum and impact on recruitment

Over the past several decades, patterns of use of opioids for the management of pain have changed significantly. In the 1980s, physicians generally avoided using opioids. Then, at the turn of the millennium opioid use increased to the point of overuse for the first decade and since then the pendulum has swung back against the use of opioids in people with chronic pain.^27^ Since 2011, the general use of prescription opioids has declined significantly to the extent that access for appropriate medical use was significantly compromised and many would say that the pendulum has swung too far against opioid use.^27–30^ This is the context within which we were trying to recruit for this study. According to Table 2, recruitment in this study may have suffered from both ends of this spectrum. On the one hand, we had many screen failures due to people already being on an opioid or having had an adverse event or previous failed trial of an opioid. This is understandable given that the study took place at tertiary care pain clinics where patients have often failed first- and second-line treatments. On the other hand, from comments noted in the phone log from people screened, at least six patients decided against participating in the study because their family physicians did not want them to take an opioid, and several others did not want to use an opioid for pain control. This, in combination with the other challenges discussed above, contributed to recruitment challenges.

Initially we were able to obtain extensions on the grants supporting the study, but after two extensions the study had to be shut down due to slow recruitment. In the end, we could not answer the major question of the study due to an underpowered sample size. Further head-to-head trials comparing methadone to a conventional opioid like morphine will have to take into account the burden of the study design and the societal perception of opioids for chronic non-cancer pain.
